# Hydrogen-Rich Saline Alleviates Kidney Fibrosis Following AKI and Retains Klotho Expression

**DOI:** 10.3389/fphar.2017.00499

**Published:** 2017-08-11

**Authors:** Jing Chen, Han Zhang, Jiachang Hu, Yulu Gu, Ziyan Shen, Linghan Xu, Xueqi Jia, Xiaoyan Zhang, Xiaoqiang Ding

**Affiliations:** ^1^Department of Nephrology, Zhongshan Hospital, Fudan University Shanghai, China; ^2^Kidney and Dialysis Institute of Shanghai Shanghai, China; ^3^Kidney and Blood Purification Laboratory of Shanghai Shanghai, China

**Keywords:** Klotho, acute kidney injury, fibrosis, hydrogen

## Abstract

**Purpose:** Acute kidney injury (AKI) is a prominent risk factor for the development of chronic kidney disease (CKD). To date, the related mechanism and effective therapy have not been rigorously explored. The present study aims to investigate the reno-protection of hydrogen-rich saline (HRS) against ischemia/reperfusion (IR)-induced AKI.

**Methods:** Adult male C57 mice were randomly allocated into three groups: Sham, IR, IR+HRS. Renal IR injury model was generated via 35 min occlusion of bilateral kidney pedicles, and then, mice were administered with different treatments intraperitoneally in various groups. After 14- or 28-day treatment, mice were perfused and the kidneys were collected following reperfusion. Many proteins were detected by western blots, including renal fibrotic proteins [a-smooth muscle actin (a-SMA), collagen I (Col I)], Klotho, the methylation of Klotho, damage-regulated autophagy modulator (Beclin-1), and microtubule-associated protein light 3-II (LC3-II). Finally, the levels of serum blood urea nitrogen (BUN) and creatinine (Cr) were measured to investigate the renal function.

**Results:** Histological data showed that the HRS treatment significantly decreased the fibrosis in renal tissues when compared with the IR group, and both of BUN and Cr were lower in the HRS group than IR group (8.9 ± 0.6 vs. 9.9 ± 0.1 mmol/l, 51 ± 6.5 vs. 60 ± 5.8 μmol/l) (*P* < 0.05). The expression of fibrotic markers, a-SMA and Col I, showed a robust increase in IR injury models than the Sham group, which was consistent with the result of Trichrome staining. However, the levels of a-SMA and Col I expression were sharply decreased in the IR+HRS group (*P* < 0.05). IR injury also enhanced LC3-II and Beclin-1 expression, but decreased Klotho level. The Klotho level was alleviated by HRS, but LC3-II and Beclin-1 were starkly enhanced in HRS group (*P* < 0.05).

**Conclusion:** HRS showed a protective effect in the prevention of renal injury and could inhibit renal fibrosis after IR injury in mice. This role of HRS might be exerted via retaining Klotho expression and activating autophagy in the kidney.

## Introduction

Acute kidney injury (AKI) causes high morbidity and mortality in acute phase, with mortality rates reaching 50% in intensive care units (Ali et al., 2007; Grams et al., 2011). It is also a prominent risk factor for the development of chronic kidney disease (CKD). Individuals cured from AKI show increased the risk of CKD, with mortality increased by 50% within 10 years (Bucaloiu et al., 2012; Wu et al., 2014). However, the complex progression from AKI to CKD is still poorly understood (Arai et al., 2016). Therefore, the related mechanism and effective therapy have not been rigorously explored (Chawla et al., 2017).

Hydrogen has protective effects on the kidney after ischemic AKI in animal studies (Li et al., 2016), although the underlying mechanism remains unexamined. It has been reported that molecular hydrogen (H2) selectively reduces cytotoxic ROS as well as reactive nitrogen species *in vitro*, so it likely exerts antioxidant and anti-inflammatory effects on multiple animal models (Fukuda et al., 2007; Ohsawa et al., 2007). In nature, H2 is ubiquitous, which minimally alters oxidoreduction and ROS-related cell signaling pathways, and avoids significant adverse effects in patients (Hayashida et al., 2008). In addition, H2, as a gas, can penetrate bio-membranes and easily diffuse into cell organelles to target intracellular inflammatory factors. H2 has already been reported to protect brain, liver, heart, and intestines from I/R injuries (Verma et al., 2015; Malik and Ferguson, 2016; Nagpure and Bian, 2016). Moreover, H2 is a known anti-inflammatory molecule in acute pancreatitis, and colon and liver inflammation (Cheng et al., 2014; Yemm et al., 2015; Han et al., 2016).

Klotho, an anti-aging protein (Kuro-o et al., 1997), is a type 1 transmembrane protein found at high levels in renal and cerebral tissues (Li et al., 2004). Recent studies have revealed that renal Klotho expression is markedly suppressed in various experimental mouse models of kidney diseases and humans with CKD, and that Klotho deficiency is associated with progression of renal fibrosis, vascular calcification, cardiac hypertrophy, and secondary hyperparathyroidism. Its extracellular domain is released into circulation in soluble form by secretases (Imura et al., 2004) and affects multiple target organs (Hu et al., 2012, 2013b). Klotho participates in cyto-protection, and prevents apoptosis, senescence, and fibrosis, and may be critical for tissue regeneration (Hu et al., 2011, 2013a). Indeed, it protects the kidney from ischemic AKI if administered right after insult occurrence in animals (Hu et al., 2010).

Autophagy is a defense mechanism protecting and maintaining normal cell functions (Levine and Kroemer, 2008; Mizushima and Komatsu, 2011; Choi et al., 2013). Altered autophagy contributes to aging as well as multiple human ailments (Choi et al., 2013; He et al., 2014). In ischemia- (Jiang et al., 2012; Ishihara et al., 2013) or cisplatin-induced (Periyasamy-Thandavan et al., 2008; Isaka et al., 2011; Kimura et al., 2011; Kaushal, 2012; Takahashi et al., 2012) AKI and unilateral ureteral obstruction, (Li et al., 2010; Ding et al., 2014) the autophagic process is observed as well. Meanwhile, reduced autophagy confers vulnerability to the kidney regarding ischemic injury and nephrotoxicity (Periyasamy-Thandavan et al., 2008; Kimura et al., 2011; Kaushal, 2012; Takahashi et al., 2012; Livingston and Dong, 2014; Cheng et al., 2015). Associations of autophagy levels with Klotho were demonstrated in previous reports, but no consistent conclusion has been obtained so far (Shiozaki et al., 2008; Iida et al., 2011; Shu et al., 2013; Lin and Sun, 2015).

Therefore, the present study aims to explore the role of hydrogen-rich saline (HRS) in protecting kidney from AKI after ischemia/reperfusion injury.

## Materials and Methods

### Hydrogen-Rich Saline

Hydrogen was dissolved in normal saline under pressure at 0.5 MPa for 7 h to reach saturation by using an appropriate apparatus (Nanobubble Technology Co., Shanghai, China). Then, the saturated HRS was refrigerated at 4°C and sterilized by γ radiation. The concentration of HRS was 0.6 mmol/L as measured by gas chromatography. HRS was prepared every week to maintain the saturated concentration.

### Animals

Male C57 mice (10 weeks old; 20–25 g; Animal Center of Fudan University, Shanghai, China), were housed in temperature- and humidity-controlled rooms, with water and chow available *ad libitum*, under a 12-h photoperiod. This study had approval from the Institutional Animal Care and Use Committee of Fudan University, and was performed according to the NIH Guide for the Care and Use of Laboratory Animals. Surgery was performed under sodium pentobarbital anesthesia, and animal pain was minimized.

### Experimental Protocols

Renal IR injury was generated via 35 min occlusion of bilateral kidney pedicles. Sham-operated animals were subjected to the same anesthesia process without blocking the blood vessels. Blood and kidney specimens were collected at two-time points (14 and 28 days) after the surgery.

Three mouse groups were set up: (1) sham animals administered physiological saline (control group, *n* = 10); (2) IR models administered physiological saline (IR group, *n* = 10); (3) IR models administered daily HRS (IR+HRS group, *n* = 10). Either saline or HRS treatments were carried out at 1 ml/kg, intraperitoneally; anesthesia by intraperitoneal injection of pentobarbital (Biomics Laibo, Beijing; 0.7 mg/kg) was performed before the operation.

### Kidney Function Assessment

Blood urea nitrogen (BUN) and Cr amounts were evaluated to reflect renal function, with commercially available kits (Sigma, United States) on a COBAS Mira chemical analyzer (Roche, Switzerland).

### Histology

Kidney tissue specimens were paraffin embedded, sectioned at 4 μm and submitted to hematoxylin and eosin (H&E) and Masson trichrome staining. The analysis was performed by light microscopy, with sample blinding. Fibrosis was quantitated in Trichrome-stained kidney sections with the Image J software (Rutkowski et al., 2013).

### Immunohistochemistry

Five-micrometer tissue sections were deparaffinized and rehydrated in graded alcohol and analyzed by the streptavidin-immunoperoxidase technique. Antigen retrieval was carried out with a microwave for 10 min in 0.01 mol/L citrate buffer (pH 6.0). After blocking in 10% normal serum for 30 min, the samples were incubated at 4°C with primary antibodies targeting a-SMA, Col I, LC3-II, and Beclin-1 (Abcam, UK; 1:100). The specimens were subsequently treated with biotinylated anti-mouse or anti-rabbit secondary antibodies for 15 min at 37°C.

### Western Blotting Analysis

According to the manufacturer’s instructions, the total protein was extracted using a commercial kit (Protein Extraction Kit, Millipore). About 30 mg protein from each sample was mixed with a loading buffer and loaded onto separate lanes on 10% sodium dodecyl sulfate-polyacrylamide gel. Proteins were electrotransferred onto polyvinylidene fluoride membranes (0.2 mm: Immun-Blot, Bio-Rad) and then immunoblotted with antibodies. The antibodies and dilution for α-smooth muscle actin (α-SMA), Col I, LC3-II, Beclin-1, Klotho, and β-actin are listed in Supplementary Table S2. The intensity of each band was quantified using the NIH Image software (Bethesda, MD, United States) and the densitometric intensity corresponding to each band was normalized against β-actin expression.

### Time RT-PCR

RNA samples from kidney were extracted using TRIzol reagent (Invitrogen, Carlsbad, CA, United States), and reverse-transcribed using PrimeScript^®^ RT reagent Kit (TaKaRa, Japan). Real-time polymerase chain reaction (PCR) was carried out with SYBR^®^ Premix ExTaq^TM^ from TaKaRa. The following primers (Sangon, China) were used: Klotho, forward 5′-CACGGCAAGGGTGCGTCCAT-3′ and reverse 5′-TCGCGCCCACGAGATGGAGA-3′; β-actin, forward 5′-GATTACTGCCCTGGCTCCTA-3′ and reverse 5′-TCATCGTACTCCTGCTTGCT-3′. Relative mRNA amounts were derived by the 2^-ΔΔCt^ method.

### Statistical Analysis

Results are expressed as mean ± standard deviation (SD) from three independent triplicate experiments. SPSS 11.0 (SPSS Inc., United States) was utilized for statistical analyses. One-way analysis of variance (ANOVA) and Student’s *t*-test were employed to assess differences among and between groups, respectively. The significance level was set at *P* below 0.05.

## Results

### Hydrogen Promotes the Recovery of Renal Function

To investigate the effect of HRS on renal damage after IR, we evaluated the serum levels of Cr and BUN (Supplementary Table S1). Mice with IR contained dramatically elevated levels of Cr and BUN compared with sham-treated group (^∗^*P* < 0.05). These increased levels were significantly reduced by HRS administration (^#^ *P* < 0.05). These findings suggested that HRS improves renal function in mice after IR injury. Serum creatinine amounts were also decreased in the IR+HRS group compared to that in IR model animals at both 2 and 4 weeks after IR (Supplementary Table S1).

### HRS Reduces the Histopathologic Damage in Mice Induced by IR

**FIGURE 1:**
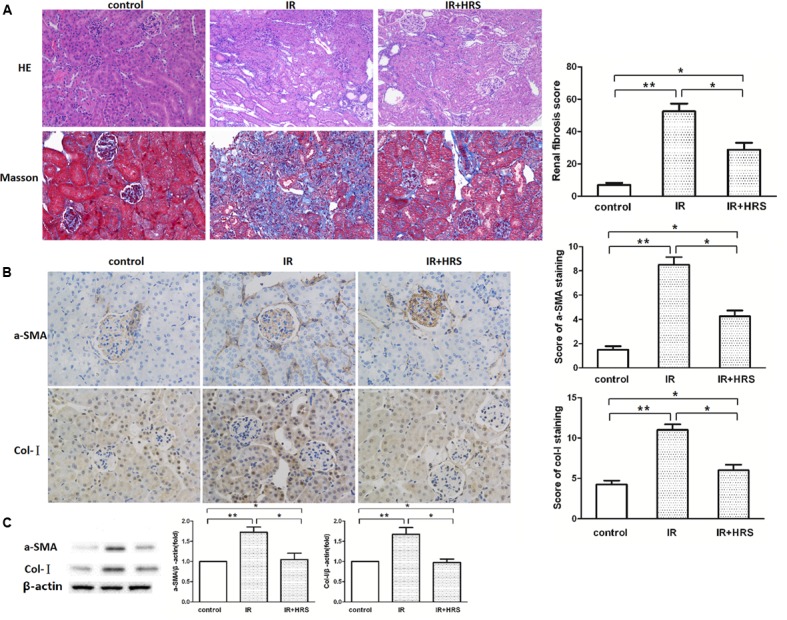
HRS Reduces the Histopathologic Damage in Mice with IR injury. **(A)** Representative photomicrographs of renal tissues submitted to hematoxylin-eosin and Masson Trichrome staining (200× magnification). Renal fibrosis scores provided semi-quantitative data of Trichrome staining findings. **(B,C)** Changes of fibrotic a-smooth muscle actin (a-SMA) and collagen I (Col I) levels by immunoblot were consistent with Trichrome staining data, with robustly increased amounts in IR models. ^∗^*P* < 0.05, ^∗∗^*P* < 0.01.

In order to verify the protective effect of HRS on kidney following renal IR injury, we analyzed kidney morphology by H&E staining and Trichrome staining after IR injury. Histological data (**Figure 1A**) demonstrated that after IR injury, glomerular collapse, tubular dilation, tubulointerstitial infiltration, and fibrosis were observed in the kidney of model animals. IR animals showed severely renal fibrosis. Changes of a-SMA and Col I determined by western blot were consistent with Trichrome staining findings, demonstrating robustly increased alteration in IR models. However, a-SMA and collagen I (Col I) expression levels were sharply decreased in the IR+HRS group (**Figures 1B,C**) (*P* < 0.05).

### Renal Klotho Level Decreased during AKI Progression to CKD

**FIGURE 2:**
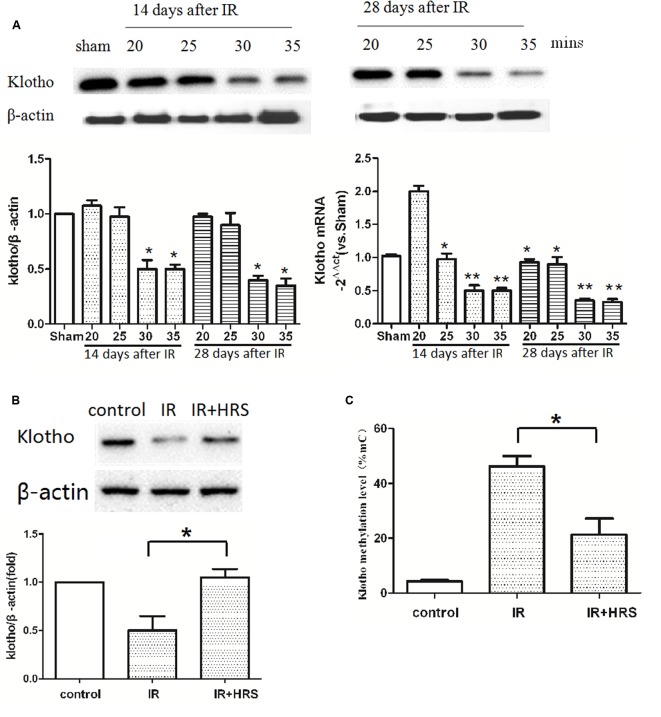
HRS unregulated Klotho expression and methylation level. **(A)** Renal Klotho levels at 14 and 28 days after IR in 20/25/30/35-min ischemia models. The protein level of renal Klotho was decreased at about 14 days after IR in 30-min ischemia models, with subsequent decrease until 28 days after the injury (^∗^*P* < 0.05). The gene expression of Klotho in 20-min ischemia models was significantly higher than the normal condition of the sham group at 14 days after IR, and this increase was abolished at 28 days after the injury **(A)** (compared with sham group, ^∗^*P* < 0.05). Both 30- and 35-min groups showed decreased Klotho gene expression at 14 days (compared with sham group, ^∗∗^*P* < 0.01). At 28 days, Klotho gene expression showed a decreasing trend in 30-min models whereas it remained at a lower level in 35-min models (compared with sham group, ^∗∗^*P* < 0.01). **(B)** The expression of renal Klotho was sharply increased in HRS group (compared with IR group, ^∗^*P* < 0.05). **(C)** The methylation level of Klotho in HRS group was decreased (compared with IR group, ^∗^*P* < 0.05).

It has been reported that Klotho reduction depends on insult severity and injury duration. To investigate the relationship between them, we detected the expression of Klotho in IR models with a different period of blockage. The protein level of renal Klotho was decreased at about 14 days after IR in 30-min ischemia models, with subsequent decrease until 28 days after the injury (**Figure 2A**) (*P* < 0.05). The gene expression of Klotho in 20-min ischemia models was significantly higher than the normal condition of the sham group at 14 days after IR, and this increase was abolished at 28 days after the injury (**Figure 2A**) (*P* < 0.01). These findings suggested mild ischemia might increase Klotho gene expression in kidney tissues in a fashion similar to preconditioned hypoxia. Both 30- and 35-min groups showed decreased Klotho gene expression at 14 days. At 28 days, Klotho gene expression showed an increasing trend in 30-min models whereas it remained at a lower level in 35-min models (**Figure 2A**). These results suggested severer insult after prolonged ischemia causes Klotho expression to persistently and irreversibly, at the protein and gene expression levels.

The severity of Klotho deficiency was further assessed at 4 weeks post-AKI. Klotho amounts (protein and mRNA) in kidney samples were decreased (**Figure 2B**) (*P* < 0.05); the animals of the IR group had slightly elevated Klotho protein expression. However, Klotho protein and mRNA expression levels were increased in the IR+HRS group. We next found that Klotho methylation was decreased (**Figure 2C**) (*P* < 0.05). These results suggested that HRS through decreasing the methylation of Klotho protects the kidney from acute ischemia.

### Effect of HRS on Autophagy-Related Proteins Levels in Kidney tissues

**FIGURE 3:**
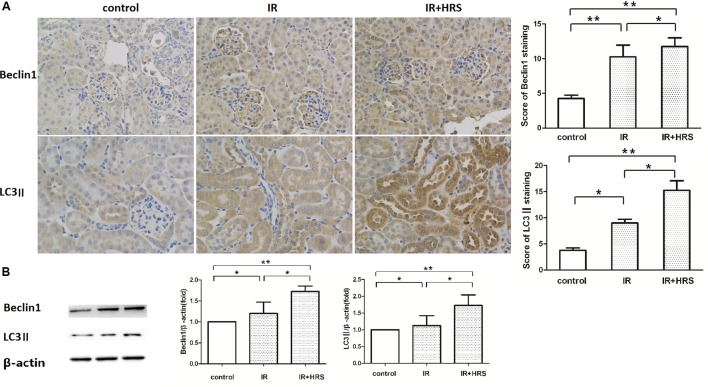
The expression of autophagy markers in these groups. **(A,B)** HRS enhanced the protein levels of autophagy markers in renal tissues of IR mice 28 days after reperfusion. Renal samples were collected 28 days after reperfusion to measure Beclin-1 and LC3-II expression levels by immunohistochemistry and western blot. LC3-II and Beclin-1 in the IR group were concurrently increased, compared to the sham group (^∗^*P* < 0.05). HRS treatment significantly elevated LC3-II and Beclin-1 expression levels compared with the IR group (^∗^*P* < 0.05).

**FIGURE 4:**
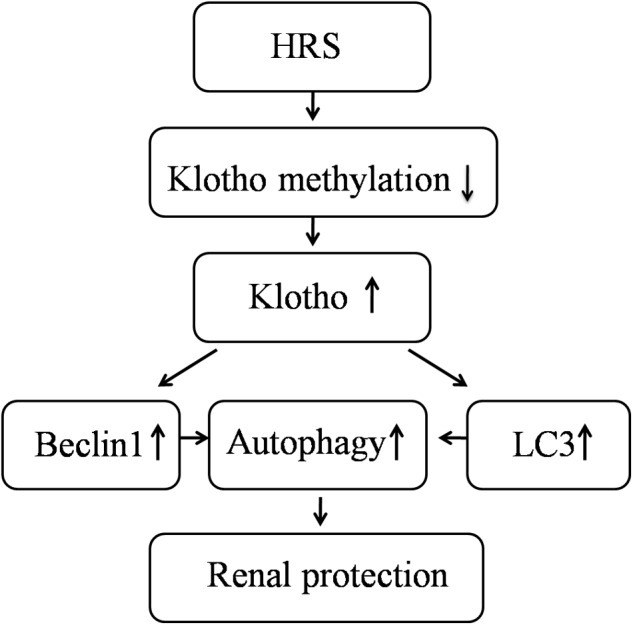
Schematic illustration of the molecular mechanisms of HRS-induced renoprotection against AKI to CKD after IR.

To further study the mechanism by which HRS reduces renal fibrosis after IR, we assessed the expression levels of proteins related to autophagy using western blot. As shown in **Figures 3A,B**, LC3-II and Beclin-1 in the IR group were concurrently increased, compared to the sham group (*P* < 0.05). In addition, HRS treatment significantly elevated LC3-II and Beclin-1 expression levels compared with the IR group (*P* < 0.05). Autophagy was up-regulated in the IR+HRS group compared with the IR group. These findings supported autophagy may play an important role in the therapeutic effect of HRS in AKI to CKD after IR (**Figure 4**).

## Discussion

This study showed HRS is able to promote renal function recovery after IR injury in mice by increasing Klotho expression, activating autophagy and inhibiting renal fibrosis. These results indicated that continuous HRS treatment was a profound therapeutic strategy for the kidney after IR injury.

Renal IR injury is commonly found in multiple clinical conditions such as renal transplantation and shock. Multiple reports suggested that hydrogen downregulates inflammatory cytokines, thus decreasing kidney IR (Shingu et al., 2010; Koning et al., 2015). However, in these studies, expression of various inflammatory cytokines and apoptosis-related proteins in the kidney tissues was only measured at 24 h after IR injury. Subsequent renal function recovery is seldom reported. Consequently, the present work evaluated long-term HRS administration for its protective impact on renal fibrosis after IR injury, exploring the related mechanisms. According to the result of histological data, we found that HRS decreased a-SMA and Col I expression. HRS could protect renal function from IR injury in the long term.

Previously published reports demonstrated that serum Cr and BUN levels in rats administered HRS after kidney IR are markedly decreased (Shingu et al., 2010; Wang et al., 2011). However, IR injury is a sustained condition. Li et al. (2016) found that the level of Cr could almost recovery at 108 h after IR, but our study exhibited that the levels of Cr and BUN dramatically elevated compared with control group at both 2 and 4 weeks after IR injury. The possible reason for this is different IR models we used. Li et al. (2016) used the rat model of renal IR injury induced by 45 min occlusion of the left renal pedicle and removal of the right kidney, but we used the model of renal IR injury induced by 35 min occlusion of bilateral renal pedicles. It was demonstrated that hydrogen only protects renal function at 24 h after IR, with no subsequent renal recovery. As shown above, we found that 28 days of continuous HRS administration promoted kidney function recovery.

The role of autophagy in IR injury is still controversial. From one perspective, autophagy can disassemble the cytoplasmic parts and intracellular organelles which are not necessary to eliminate damaged organelles and maintain the balance of protein synthesis (Guan et al., 2015). However, it has also been reported that uncontrolled autophagy can lead to the irreversible demise of cells (Huang et al., 2015). Our study explored the role of autophagy in regulating the process of renal fibrosis after IR injury. We assessed the expression levels of proteins related to autophagy using western blot, including LC3-II and Beclin-1. As shown in results, the expressions of these proteins were increased in IR group, compared to sham group. In addition, HRS treatment significantly elevated LC3-II and Beclin-1 expression levels compared with the IR group. Autophagy was up-regulated in the IR+HRS group compared with the IR group. These findings supported autophagy may play an important role in the therapeutic effect of HRS in AKI to CKD after IR.

Klotho deficiency contributes to kidney fibrosis (Takeshita et al., 2002; Doi et al., 2011; Satoh et al., 2012; Zhou et al., 2013; Eren et al., 2014). Previous reports demonstrating improved kidney function protection in Tg-Kl with elevated kidney and blood Klotho after insult (Hu et al., 2010, 2011; Panesso et al., 2014) does not detect which of the renal membrane and blood Klotho is more significant in reno-protection. Treatment with exogenous Klotho enhanced kidney function recovery while reducing fibrosis, confirming that soluble Klotho is active. Previous studies have demonstrated that HRS treatment protects the renal tissue from IR injury by suppressing the levels of inflammatory mediators, but how the level of renal-protective protein Klotho changed was not known. Thus, we assayed Klotho gene methylation levels and its protein levels. The results showed that Klotho was sharply downregulated in IR group; however, its expression was upregulated after the HRS treatment. These findings suggested that HRS protective effect on kidney injury might be mediated by upregulating Klotho expression and its downstream signaling pathway.

This study has certain limitations. First, this is a descriptive study, and we observed the changes of Klotho level, autophagy level and renal fibrosis level at the end point of the experiments. Though those pathological events were detected simultaneously, we did not provide direct evidence to prove their relationship. Further researches will focus on strategies to manipulate autophagy and Klotho and their effects on HRS protection in kidney fibrosis. Second, an increased level of LC3-II and Beclin-1 at a steady state does not necessarily indicate that autophagy is activated, as autophagy is a dynamic cellular process. Further researches need to be included to monitor autophagy dynamics accurately and to examine the effects of HRS on autophagy.

In summary, our study demonstrated that HRS could attenuate renal IR injury in a mice model and promote autophagic activity in the process of AKI to CKD. Perhaps, the epigenetic regulation of Klotho is associated with these effects. Compared with hydrogen gas, H2 saturated saline solution (HRS) is safe and easy to administer. Animal studies have shown that HRS has protective effects on brain, liver, heart, and kidney. Li et al. (2016) have reported that HRS has therapeutic effects on renal IR injury after 108 h reperfusion. Based on these concerns, the HRS treatment is not supposed to affect the normal physiological condition. In addition, the major aim of this project is to address the effect of HRS on kidney fibrosis after IR. We have not observed any side effect of this treatment so far. Therefore it is a safe and effective therapeutic intervention. Overall, the current work showed continuous hydrogen administration could retain Klotho expression and protect renal function. Although the mechanisms involved in hydrogen’s protective role remain to be fully elucidated, peritoneal injection of hydrogen could represent an easy to use, safe, cost efficient and effective novel approach for future kidney injury protection.

## Author Contributions

Conceived and designed the experiments: JC. Performed the experiments: JC and HZ. Analyzed the data: JC and XZ. Contributed reagents/materials/analysis tools: JH, LX, XJ, YG, and ZS. Wrote the manuscript: JC, XZ, and XD.

## Conflict of Interest Statement

The authors declare that the research was conducted in the absence of any commercial or financial relationships that could be construed as a potential conflict of interest.
