# Supplementary material for: Hydrogen-Rich Saline Alleviates Kidney Fibrosis Following AKI and Retains Klotho Expression
Source: Front Pharmacol. 2017 Aug 11;8:499. doi: 10.3389/fphar.2017.00499 (PMC5554490; doi:10.3389/fphar.2017.00499)
Supplement: Supplementary file 1 [file Table_1.pdf]

	14 days after AKI			28 days after AKI		
	sham	IR	HRS +IR	sham	IR	HRS +IR
Scr(μmol/l)	28.3±3.4	48±6.1*	42±5.4#	28±3.2	60±5.8*	51±6.5#
BUN(mmol/l)	4.7±0.5	6.2±0.9*	5.9±0.7#	5.8±07	9.9±1.1*	8.9±0.6#
UACR(μg/mg)	4.5±1.3	7.5±1.7*	7.1±1.6#	4.9±15	8.3±1.3*	7.9±1.7#

S1 The effect of HRS on renal damage after IR, we evaluated the serum levels of Cr and BUN (S1). IR dramatically elevated Cr and BUN levels compared with sham-treated group(*$P<0.05$). The increased Cr and BUN levels were reduced by HRS administration (#$P<0.05$).

Table S2. The antibodies for Western blot.

Protein	Species	Antibody	Source	Dilution
**α-SMA**	Rat	Rabbit polyclonal to α-SMA	Abcam, Cambridge, MA, USA	1:1000
**Col-I**	Rat	Mouse monoclonal to Col-I	Santa Cruz Biotechnology, Dallas, Texas, USA	1:1000
**LC3-II**	Rat	Rabbit polyclonal to LC3-II	Abcam, Cambridge, MA, USA	1:1000
**Klotho**	Rat	Rabbit polyclonal to Klotho	Abcam, Cambridge, MA, USA	1:3000
**β-actin**	Rat	Mouse monoclonal to actin	Abcam, Cambridge, MA, USA	1:5000
